# Cytomegaloviruses and Macrophages—Friends and Foes From Early on?

**DOI:** 10.3389/fimmu.2020.00793

**Published:** 2020-05-12

**Authors:** Sebastian Baasch, Zsolt Ruzsics, Philipp Henneke

**Affiliations:** ^1^Institute for Immunodeficiency, Center for Chronic Immunodeficiency (CCI), Medical Center - University of Freiburg, Faculty of Medicine, University of Freiburg, Freiburg, Germany; ^2^Center for Pediatrics and Adolescent Medicine, University of Freiburg, Freiburg, Germany; ^3^Institute of Virology, Medical Center - University of Freiburg, Faculty of Medicine, University of Freiburg, Freiburg, Germany

**Keywords:** macrophage, monocyte, CMV (cytomegalovirus), innate immunity, pathogen-host coevolution, mucosal immune barrier, virus-host adaptation, macrophage heterogeneity

## Abstract

Starting at birth, newborn infants are exposed to numerous microorganisms. Adaptation of the innate immune system to them is a delicate process, with potentially advantageous and harmful implications for health development. Cytomegaloviruses (CMVs) are highly adapted to their specific mammalian hosts, with which they share millions of years of co-evolution. Throughout the history of mankind, human CMV has infected most infants in the first months of life without overt implications for health. Thus, CMV infections are intertwined with normal immune development. Nonetheless, CMV has retained substantial pathogenicity following infection *in utero* or in situations of immunosuppression, leading to pathology in virtually any organ and particularly the central nervous system (CNS). CMVs enter the host through mucosal interfaces of the gastrointestinal and respiratory tract, where macrophages (MACs) are the most abundant immune cell type. Tissue MACs and their potential progenitors, monocytes, are established target cells of CMVs. Recently, several discoveries have revolutionized our understanding on the pre- and postnatal development and site-specific adaptation of tissue MACs. In this review, we explore experimental evidences and concepts on how CMV infections may impact on MAC development and activation as part of host-virus co-adaptation.

## Introduction

The human body harbors diverse communities of microorganisms, in particular bacteria and fungi colonizing the outer and inner surfaces of the body (microbiome), as well as latently infecting viruses (virome) (1). This ecosystem is subject to influences, e.g. nutrient supply, interspecies competition and diffusible immunological effector molecules. At the same time, microbiome and virome shape host immunity via direct interaction with immune and non-immune cells, and indirectly, e.g. via secreted metabolites (2). In contrast to extracellular bacteria and fungi, which are largely controlled on the population level, viruses can be expected to rely on reciprocal adaptations with the individually infected host cell. The genus of cytomegaloviruses (CMVs), which belong to the subfamily of betaherpesvirinae, have co-evolved with their mammalian hosts for millions of years (3). In humans, infection with human CMV (HCMV) usually occurs in the first months of life, although infection has been pushed toward later life in highly industrialized societies (4–6). Therefore, HCMV is part of a “physiological” virome in immunocompetent individuals. Infants are infected via smear infections or via HCMV-containing milk as seropositive mothers reactivate HCMV locally (7) and transmit the virus to their children in more than 30% of cases (8).

CMVs have co-evolved with their specific hosts. Therefore, cross species infection models to study virus-host interactions are not available *in vivo*. Murine CMV (MCMV) and HCMV share only 45% of their genes (9), but have many similarities in cell tropism and immune modulatory properties. Hence, MCMV infection of mice is regarded as a useful experimental model to understand HCMV pathology (10). In the subsequent text we will use the abbreviation “CMV” in the case of general statements and if features are shared by the CMVs, which were studied.

Intraperitoneal and subcutaneous (foot pad) infections have provided valuable information on MCMV biology in the complex *in vivo* situation. However, since breast milk and saliva are regarded as important HCMV and MCMV sources, intragastric and intranasal infections have more recently been exploited (11). HCMV may infect cells of the mouth/upper gastro-intestinal tract, or it may reach the intestine. Moreover, HCMV may infect the respiratory tract via aspiration of virus containing milk. MACs and their potential progenitors, circulating monocytes, are well-known target cells for CMV (12–16). In the barrier tissues of intestinal and respiratory tracts, MACs represent the most abundant immune cells. However, tissue resident MACs are highly heterogeneous and undergo age specific changes during the individual host development, with respect to their origin and the tissue they inhere (17). For example, lamina propria MACs (LpMAC) in the intestine and microglia in the CNS represent two extremes with and without replenishment by monocytes, respectively. Models on how the phenotypic and functional MAC diversity impacts on CMV infections and vice versa are still in infancy.

In this review we focus on the ability of MACs to recognize CMV early after infection, and the known cellular consequences of infected MACs with regard of cytokine production and polarization. We summarize mechanisms of how CMV exploits monocyte influx and discuss potential consequences in putative target tissues. We propose that early CMV infections train the monocyte-macrophage-axis and are therefore beneficial in the immunocompetent host. Finally, we highlight the central role of monocytes and MACs in CMV infection serving as latent reservoirs and reactivation sites.

## CMV recognition by macrophages and monocytes

The high frequency of tissue MACs in CMV entry sites, e.g. the lamina propria (intestinal tract) or alveolar spaces, allows for a potent response to epithelial barrier disruption and invasion of microorganisms, such as bacteria, or viruses. In order to cover a huge variety of pathogens with distinct extracellular or intracellular lifestyles, MACs and monocytes are equipped with pattern recognition receptors on plasma and endosomal membranes and in the cytosol. Together, these receptors recognize conserved microbial molecules or alterations in host structures, such as nucleic acids occurring at atypical sites. The engagement of pattern recognition receptors leads to the formation of cytokines, which are suited to initiate an appropriate immune response. During viral infections, type I interferons (IFN I) play an important role in creating a hostile cellular environment for viral replication and spread (18). Accordingly, mice deficient in the IFN I receptor (IFNAR^−/−^) succumb to CMV infection (19). Furthermore, inflammasome-dependent secretion of interleukin 18 (IL-18) augments NK-cell function in MCMV infections (20).

### CMV and Toll-Like Receptors

Upon ligand binding Toll-like receptors (TLRs) transduce signals via the cytosolic adapter molecule myeloid differentiation primary response 88 (MyD88). In this respect, TLR3 is an exception, since it uses TIR-domain-containing adapter inducing interferon-β (TRIF) and TRIF-related adaptor molecule (TRAM) as sole adapters (21). To induce IFN I transcription, dimerization of transcription factors interferon regulatory factor (IRF) 3 (through TLR3-TRIF) and/or IRF7 (through TLR9-MyD88) is essential. Accordingly, peritoneal MAC from IRF3 and IRF7 double knockout mice do not produce IFN-β when infected with MCMV (22). The role of upstream MyD88 in IFN I production in MCMV infection was confirmed in several studies (23–25). A loss-of-function frameshift mutation in TRIF increases susceptibility and diminishes circulating IFN I in MCMV infection (26). Moreover, bone marrow cells from mice with a combined deficiency in MyD88 and TRIF, showed an impaired IFN I formation in MCMV infection *in vitro*. However, residual IFN I formation in these cells suggests the existence of a TLR-independent pathway (27).

**Figure 1:**
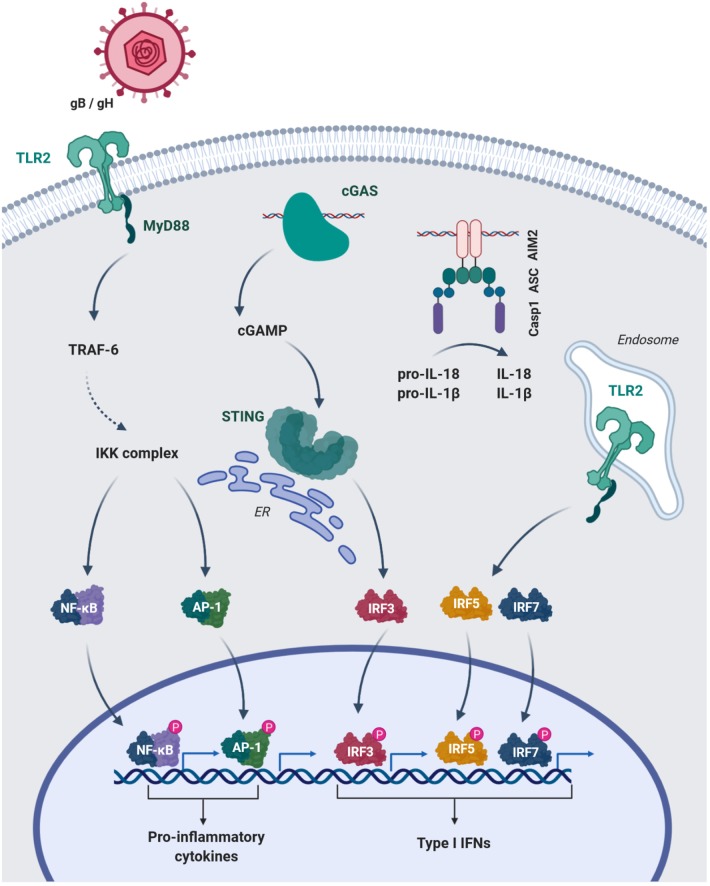
HCMV glycoproteins B and H (gB/gH) engange TLR2 of MAC on the surface and activate NFκB and AP-1 mediated transcription of pro-inflammatory cytokines. The cytosolic sensors cGAS and AIM2 recognize CMV-DNA. cGAS produces the signaling mediator cGAMP, which leads to STING activation and IRF3-dependent type I IFN transcription. Engagement of DNA by the HIN domain of AIM2 leads to interaction with the adaptor ASC (PYD-PYD) and subsequent recruitment of pro-caspase 1 via their CARD domains. Activated caspase 1 (Casp1) can cleave the pro-forms of IL-18 and IL-1β converting them into their mature bioactive forms. Additionally, inflammatory monocytes can uptake DNA viruses into endosomal compartments and induce type I IFN via IRF5 and 7. TRAF-6, TNF receptor associated factor-6; IKK-complex, IκB kinase-complex; AP-1, Activator protein-1; ER, endoplasmatic reticulum; ASC, Apoptosis-asspciated speck-like protein containing a CARD; PYD, pyrin domain.

The strictly intracellular lifestyle of CMV requires expression of host cell receptors that provide docking sites for viral ligands and facilitate cellular entry. Complexes of the CMV glycopoteins B and H (gB, gH) mediate host cell entry (28). Although, the entry-mediating host receptors for these protein complexes are still controversial (29), TLR2, which is expressed on the cell membrane, is known to interact with gB/ gH (30). TLR2 binding drives HCMV-induced nuclear factor kappa-light-chain-enhancer of activated B cells (NFκB)-dependent production of inflammatory cytokines in MACs (31) (Figure 1) and mediates the control of CMV in immunocompromised humans and mice (32, 33). Interestingly, TLR2-dependent IFN I production has been found to be specific for inflammatory monocytes (27) (Figure 1), while dendritic cells did not mount an IFN I response through TLR2 (27).

In addition, endosomal TLRs, e.g. TLR 3, 7 and 9, are involved in MCMV recognition, since a missense mutation in their adapter UNC-93B leads to substantially decreased formation of interferon gamma (IFN-γ), IL-12, tumor necrosis factor (TNF) and IFN I in the plasma of mice after intraperitoneal MCMV infection (34).

Although the cell specific contribution of individual TLRs remains unclear, interaction of MCMV with TLR2 on monocytes and MACs may contribute to the rapid mounting of inflammatory and antiviral mediators in case of viremia. In contrast, TLR9 in dendritic cells seems to be crucial for IFN I in later stages of infection (24, 34–37).

### CMV and Cytosolic Sensors

The observation that MACs, which accumulate DNA in phagosomes due to a DNAse II-deficiency, induce interferon-mediated anemia, which was reversed by deletion of *IFNAR* (38), suggested a TLR9-independent DNA sensor in the cytosol. Moreover, MACs deficient in MyD88, TRIF and mitochondrial antiviral signaling (MAVS) protein maintained a IFN I response in MCMV infection (39). The identification of the stimulator of interferon genes (STING) (40, 41) and cyclic guanosin monophosphate-adenosine monophosphate synthase (cGAS) (42), the upstream sensor for cytosolic DNA, provided mechanisms for the recognition of self and microbial DNA, e.g. from herpesviruses (43, 44). Tegtmeyer et al. recently demonstrated the importance of cGAS-STING-signaling in MCMV infection (45). STING-mediated IFN I was induced as early as 4 h post infection (hpi) (45). Kupffer cells, the liver resident MACs, were a main source of STING-dependent IFN I and restrained viral dissemination to the lymphnodes (45). These results support data generated *in vitro* in immortalized bone marrow-derived MACs, which responded to MCMV infection with IFN-β in a cGAS-STING-dependent fashion (46). Accordingly, HCMV induces IFN I in human monocyte-derived MACs via cGAS and STING (47) (Figure 1).

The DNA-dependent activator of IFN-regulatory factors (DAI) has been identified as another cytosolic sensor of nucleic acids (48). DAI interacts with TANK binding kinase 1 (TBK1) and IRF3, suggesting IRF3-mediated transcription of IFN I (48). Moreover, DAI can activate NFκB via RHIM/RIP1 and 3 recruitment (49, 50). However, the importance of DAI-mediated activation of NFκB and IRF3 in the IFN I response to cytosolic DNA seems marginal (51). Moreover, DAI has been recently challenged as a specific DNA sensor, since it was shown to interact with genomic RNA of influenza A virus (52) and with newly synthesized MCMV RNA (53), ultimately leading to necroptosis of fibroblasts (52, 53). This is in line with previous studies, where infection with MCMV, lacking the viral inhibitor of RIP activation (vIRA/m45) (54–56), induced DAI-RIPK3-dependent necroptosis in MACs (57). These findings support a role of DAI-dependent cell death induction in infected cells, rather than in direct innate immune activation.

The interaction of CMV with intracellular sensors appears to induce cellular activation beyond IFN I. A prominent example is the engagement of the absent in melanoma 2 (AIM2) by MCMV DNA in MACs, which leads to the maturation of IL-1β and IL-18 via caspase 1 cleavage (22) (Figure 1). Inflammasome activation and IL-18 secretion is essential for NK cell activation, as the formation of IFN-γ by these cells is reduced in ASC^−/−^ and AIM2^−/−^ mice (22).

Overall, MACs recognize CMV particles and form IFN I to restrict viral spread within hours after infections (45, 58). *In vitro* data generated in MACs further suggest that they induce NK-cell recruitment and IFN-γ production via inflammasome activation during the early response (Figure 1). On the other hand, MCMV and HCMV have evolved numerous strategies to evade the IFN I response (59–62). As an example, the MCMV protein M35 antagonizes IFN I induction downstream of STING and TLRs and thus ensures MCMV replication in MACs (46) (Table 1).

**Table 1:** Genes of MCMV and their HCMV homologs in this review.

**Gene**	**m129 (Mck2)**	**M36 (vICA)**	**m139, m140, m141**	**M35**	**M45 (vIRA)**
MAC specific	Partly	Yes	Yes	Partly	No
Function	Viral CC chemokine homolog to attract monocytes; essential for *in vitro* infection of MAC in MCMV	Inhibition of caspase 8 activation (apoptosis); inhibition of innate immune response of MAC; essential for *in vitro* replication in MAC	Capsid formation in MAC	Interference with NFκB-dependent IFN I transcription; essential for *in vitro* replication in MAC	Inhibition of necroptosis (inhibition of RIP3 activation); activation of NFκB (early); inhibition of NFκB essential modulator (NEMO) (late)
HCMV ortholog/ homolog	Yes (UL128)	Yes (UL36)	No	Yes (UL35)	Yes (UL45); not a functional homolog
Literature	(63–65)	(66–68)	(69, 70)	(46)	(57, 71, 72)

## Cytomegalovirus Affects Dynamics of Monocyte Recruitment to Infected Tissues

As outlined above, MACs can be a major source of IFN I in CMV infections. IFN I induce CCL2 and, to a lesser extent, CCL7 and 12 in the liver and the bone-marrow in MCMV infections (73, 74). A gradient of CCL2, which binds to its receptor CCR2, facilitates the egress of inflammatory monocytes from the bone marrow into the blood stream and the entry into infected tissues (75–79). Notably, all CMV species encode for viral chemokines located directly downstream to their major immediate early locus, corresponding to the UL128-UL131A region in HCMV. In MCMV, the viral chemokine is encoded by the m131 and m129 ORFs, which are fused by splicing. The resulting transcript encodes for a single protein called MCK-2 (80), which cooperates with CCL2 to attract monocytes (63) (Table 1). In HCMV, the chemokine homolog is encoded by the UL128 gene. The chemokine function of this gene product has not been well-studied yet. This may change in the near future by exploiting primate CMV models (81).

At the site of infection, Ly6C^hi^ inflammatory monocytes can differentiate into monocyte-derived dendritic cells or MACs, which express the inducible nitric oxide synthase iNOS (82, 83). Nitric oxide formed by inflammatory monocytes inhibits CD8^+^ T-cells and thus modulates adaptive immunity in MCMV infection (84). Additionally, recruitment of inflammatory monocytes increases the number of NK-cells via CCL3 in the liver (85). NK-cells contain the T-cell response by killing of infected antigen presenting cells (86). This process contributes to MCMV persistence (86).

In a foot pad infection model with MCMV, a second subset of monocytes, Ly6C^lo^ CX_3_CR1^hi^ patrolling monocytes, are rapidly recruited (64). Intravital microscopy revealed that patrolling monocytes crawl along the inner lining of blood vessels (87) to remove particles inside the vessel lumen (88). Accordingly, patrolling monocytes may acquire MCMV from Tie2^+^ endothelial cells (89) and outmatch inflammatory monocytes as primary targets of MCMV. They harbor viral DNA and serve as vehicles to disseminate MCMV to the spleen and salivary gland (64). However, the roles of the chemokine receptor CX_3_CR1, which is highly expressed on patrolling monocytes, and MCK-2, which supports the recruitment of patrolling monocytes, are controversial in the context of viral spread (64, 90). In contrast to an earlier study (64), Farrell et al. did not find significant differences in MCMV salivary gland titers between CX_3_CR1-deficient and control mice after foot pad infection (90). Furthermore, MCK-2-deficient MCMV spreads in similar magnitude as wt-MCMV to the salivary gland 5 days after lung infection. A significant role of MCK-2 in MCMV dissemination was found only late, i.e. 10 days post infection (90). These findings highlight the value of kinetic experiments especially in elaborate *in vivo* experiments.

Collectively, MCMV infection leads to the recruitment of inflammatory and patrolling monocytes. While this appears to assist viral spread during initial infection, the monocyte influx changes the tissue-specific cell composition and might thus ultimately affect adaptive immunity and tissue integrity.

## Monocyte Contribution to Tissue Macrophages in Steady State and Infections

It has been appreciated for a long time that circulating monocytes can be the direct progenitors of tissue MACs (91). However, in the last decade, substantial heterogeneity of MACs in different organs with respect to origin, renewal and immunophenotype has been uncovered (92–94). Resident MACs are seeded already in the embryo, either directly from the yolk-sac or via fetal liver-derived monocytes (17, 95). Postnatally, with increasing age and adaptation to the outer world, monocytes replenish MACs of the heart (96), the skin (97) and the intestine (98) even in steady state. This situation changes during inflammation or infection, when monocytes are recruited in great numbers also to other tissues (17, 99).

The depletion of MACs, e.g. via lytic infection of CMV, opens niches in the resident tissue MAC pool, which may be filled by invading monocytes (100). In mice, Ly6C^hi^ monocytes give rise to MACs in the skin (97). Patrolling monocytes, on the other hand, fail to populate the intestine after depletion of CD11c^+^ MACs (101). Accordingly, inflammatory and not patrolling monocytes are considered to be the source of tissue MACs under described conditions. However, it seems highly context and tissue dependent, whether monocyte-derived MACs fully adapt and turn into long lived resident MACs or act as “transitory” cells (102). Monocytes cease to engraft into some tissues once inflammation has resolved (103–105). While they poorly perform tissue specific functions to prevent pulmonary alveolar proteinosis in the lung (106), they successfully replace and functionally restore resident cells in other organs, e.g. Kupffer cells in the liver (107). Therefore, origin and time of tissue invasion can impact on MAC function. Interestingly, monocyte-derived MACs may also show context and tissue-specific functional properties during inflammation (108, 109). As examples, monocytes ensure tissue regeneration after skeletal muscle injury (109), yet they show high inflammatory activity during DSS-induced colitis (108). This suggests that fine tuning of MAC function is largely influenced by local cues of the target tissue (110).

In summary, CMV infections and subsequent monocyte recruitment most likely have tissue specific consequences for the resident MAC population and therefore function, which can be either beneficial or deleterious. Thus, all organs, which are targeted in CMV infection deserve individual investigation.

## Macrophages—a special target for CMV?

MACs are defined by morphology, phenotype and function, i.e. phagocytosis and cytokine secretion (111). Tissue MACs are terminally differentiated, however they retain plasticity to react on changing environmental cues, like those induced in infections (112). Next to their prominent role as a first line of defense, MACs in barrier tissues also bear central functions to maintain an anti-inflammatory state in homeostasis. Attempts to grasp the response capacity of MACs to different stimuli have led to a conceptionally useful, but oversimplifying view of pro-inflammatory (M1) and anti-inflammatory, or regulatory (M2) MAC polarization states (113).

Interestingly, polarization of MACs toward either a pro-inflammatory or regulatory state before exposure to HCMV and MCMV alters their susceptibility to infection *in vitro* (114, 115). With respect to putative MCMV virulence factors, MCK-2 deficiency limits CMV infectivity of MACs *in vitro* (65) and *in vivo* (116). MCK-2 appears to be incorporated in infectious particles via binding to virion glycoproteins gH and gL. Since gH/gL is curtail for cell entry, MCK-2 binding to gH/gL has a potential to modulate viral tropism (65) (Table 1). In contrast to the relatively clear *in vitro* phenotype, MCK-2 deficiency does not show a strong tropism phenotype *in vivo* (117). In HCMV, the MCK-2 homolog UL128 participates in the formation of the alternative gH/gL envelop glycoprotein complex, which also appears to influence viral host cell tropism (118).

HCMV can induce inflammatory transcriptional programs in MACs as soon as 4 hpi, with upregulation of genes of the ontology “Anti-viral response” (119). Within 24 hpi inflammatory cytokines are secreted (114) involving NFκB, phosphoinositide 3-kinase (PI3K) (120) and IRF signal transduction. The interplay of NFκB and PI3K seems necessary for early transcription of inflammatory cytokines, e.g. TNFα, but also anti-inflammatory cytokines, e.g. IL-10 (120). Accordingly, MCMV induces IL-10 production in peritoneal MACs leading to downregulation of MHCII (121). HCMV and rhesus CMV encode for a viral IL-10 homolog, which reduces migration of dendritic cells to the lymph node, as well as T-cell activation (122, 123). On the other hand, IL-10 producing CD4^+^ T-cells are induced via IFN I signaling in MACs during MCMV infection (124). IL-10 dampens inflammatory cytokines, such as IFN-γ and IL-6, attenuates tissue damage after MCMV infection (125, 126) and promotes persistence of infection in the salivary gland (127). Finally, TGFβ is secreted by infected human fibroblasts and in rat splenocytes after infection with the respective CMV species (128, 129). Thus, there is strong evidence that CMV induces immunoregulatory cytokines in MACs in addition to viral IL-10, e.g. in HCMV.

CMV inhibits apoptosis and necroptosis in MACs via its proteins pM36 (66) and pM45 (57), respectively (Table 1). The M36 gene is conserved among all CMVs and encodes for a cytosolic protein, binds to and blocks the activation of caspase-8 (66). Mutants lacking the M36 gene fail to inhibit apoptosis, show poor viral growth in MAC cell cultures, and loose *in vivo* fitness (67, 130, 131). vIRA/m45 and the cell death regulator vICA/M36 (66, 132) are essential for CMV replication in MACs. After intraperitoneal infection F4/80^+^ MACs seeded MCMV into the blood and brown adipose tissue, while CD11c^+^ myeloid cells, which can be expected to comprise dendritic cells, MACs and monocytes, were necessary for dissemination to the salivary gland after lung infection (133, 134). These findings indicate a migratory character of otherwise resident MACs. Interestingly, MCMV mutants lacking M36 (ΔM36) cannot disseminate after peripheral infection (67), and they grow normally in most of the cell types *in vitro* except for MACs. The growth impairment of MCMV mutants lacking M36 in mice with a defect adaptive immunity was rescued by the depletion of MACs. Accordingly, activated MACs were sufficient to impair ΔM36 growth in normally permissive MEFs *in vitro*. This could be reverted by caspase inhibition. TNFα from activated MAC synergized with IFN-γ in MEFs to inhibit ΔM36 growth. Hence, the altered ΔM36 growth in MAC and probably the altered virulence of this mutant does not reflect a defect in tropism, but rather a defect in the suppression of innate immune mediators secreted by infected and/or bystander MACs (67). The vICA in HCMV is encoded by the UL36 gene. The protein pUL36 also binds to pro-caspase-8, inhibits apoptosis and allows for viral replication in THP-1 cells (135). *In vivo* studies on the function of UL36 are limited due to the strict host specificity of CMVs. However, the cloning of UL36 into ΔM36 MCMV completely rescues the viral function both *in vitro* and *in vivo* (68). This indicates functional conservation of vICA in MCMV and HCMV.

Similar to M36, the complex of the products of MCMV genes m139, m140, and m141 is dispensable for the viral growth in fibroblasts, but it is essential for lytic MAC infection (66, 69) (Table 1). The products of these genes aid efficient capsid formation, which is apparent only in MACs (70). The underlying mechanism is not clear. It was proposed that the complex of pm139/pm140/pm141 influences cell type specific regulation of transport processes, which are important for assembly of infectious particles (69).

Together, cytokines formed by CMV-infected MACs, such as IFN I, TNFα and IFN-γ, help to contain viral infection (45, 58, 136), while viral and host IL-10 ensure replication and persistence of CMV. Simultaneously, IL-10 maintains a tolerogenic environment, which prevents tissue damage and may benefit the host during CMV infection. Regulatory or “unprimed” MACs can be considered to be more susceptible to CMV infections. This and the notion that neonatal innate immune cells produce lower amounts of IFN I (137), may explain, why newborn infants shed HCMV in higher concentrations than adults in primary HCMV infection (138). Furthermore, CMVs encode viral gene products (Table 1) to specifically target MACs and modulate their functions. This is decisive for viral dissemination and confers a central role to MACs in CMV infections.

## CMV infection of the respiratory tract, the intestine and the CNS

In the lung, a potential CMV entry site, two major MAC types can be discriminated: Alveolar MACs (aMACs), which reside in the alveolar space to safeguard the lung from inhaled particles or pathogens (139), and interstitial MACs (iMACs) (Table 2). Fetal liver-monocytes colonize the lung to differentiate and mature into aMACs in the first week of life (140). Under steady state conditions, the aMAC population does not receive a monocyte influx (140, 157). IMACs are a heterogeneous population of at least two sub-populations (145–147). One subset bears significant self-renewal properties, whereas the other is constantly replenished by patrolling monocytes (146), which is unique for tissue resident MACs. Under steady state conditions, iMACs constantly produce IL-10, which alters DC function and maintains regulatory T-cells (104, 108, 146, 148–150, 158–160). At the same time, aMAC secrete TGFβ to induce differentiation of naïve T-cells into FoxP3^+^ regulatory T-cells (141, 161, 162). Thus, both aMACs and iMACs contribute to an anti-inflammatory tolerogenic environment in homeostasis (141, 142, 148, 149, 161, 162). AMACs are primarily targeted after intranasal infection with MCMV and their depletion leads to higher viral burden (163). After infection Ly6C^hi^ monocytes infiltrate the lung in high numbers and are infected too (163). However, data to further define the consequences of monocyte recruitment, i.e. the fate of infected and uninfected monocytes, in intranasal MCMV infections are missing. Interestingly, inflammatory monocytes are able to enter the tissue and traffic to the draining lymph nodes (164), a sequence also described after intranasal MCMV infection (133).

**Table 2:** MAC heterogeneity in CMV target tissues (steady state).

**MAC**	**Alveolar MACs**	**Interstitial MACs**	**Lamina propria MACs**	**Microglia**	**CNS-associated MACs**
Localization	Lung: Inside alveoli	Lung interstitium: 1. Alveolar interstitium/ nerves 2. Bronchial interstitium/ blood vessels	Intestine: Lamina propria	Brain parenchyma	CNS-interfaces: 1. Meninges, 2. Perivascular space, 3. Choroid plexus
Immunophenotypic markers	F4/80^+^ CD64^+^ CX3CR1^−^ MerTK^+^ CD11b^lo^ CD11c^+^ SiglecF^+^	1. F4/80^+^ CD64^+^ CX3CR1^++^ CD206^−^ Lyve-1^lo^ CD11c^+^ MCHII^hi^ 2. F4/80^+^ CD64^+^ CX3CR1^+^ CD206^+^ Lyve-1^hi^ CD11c^lo^ MHCII^lo^	F4/80^+^ CD64^+^ CX3CR1^+^ MHCII^+^	F4/80^+^ CD64^+^ CX3CR1^+^ MerTK^+^ CD206^−^ CD45^lo^ Iba1^+^	F4/80^+^ CD64^+^ CX3CR1^+^ MerTK^+^ CD206^+^ Lyve-1^+^ (1., 2.) CD45^lo/hi^ CD36^+^ (2.) Iba1^+^
Ontogeny	Embryonic (fetal liver)	Definitive hematopoiesis	Definitive hematopoiesis and embryonic (yolk sac)	Embryonic (yolk sac)	Embryonic (yolk sac, fetal liver) and definitive hematopoiesis
Monocyte replenishment	No	Yes; inflammatory and patrolling monocytes	Yes; microbiota dependent	No	Partial turnover (choroid plexus)
Function	Phagocytosis of surfactant, apoptotic cells and inhaled particles; TGFβ production; maintenance of tolerance against allergens	IL-10 formation; prevention of type 2 response to inhaled allergens; antigen presentation, regulation of T-cell response	Phagocytosis; maintenance of regulatory T cells; epithelial cell renewal;	Phagocytosis; supply of neurotrophic factors; synaptic pruning; guidance of developing vasculature	Filtering of cerebrospinal fluid; immune surveillance; regulation of blood-brain barrier permeability
Literature	(105, 139–144)	(145–149)	(98, 104, 108, 150, 151)	(152, 153)	(154–156)

In MCMV latency, the infection may be reactivated in immunosuppressive conditions also in the lung (165). Thus, monocytes (64, 166) could carry CMV to the lung, where they can reactivate the virus upon differentiation into iMACs. Accordingly, CMV may exploit the physiological recruitment of patrolling monocytes (167–169), which serve as vehicles in MCMV infections (64).

Intestinal LpMACs represent the largest MAC subset in the mouse (170) (Table 2). They form a dense network close to the basal site of epithelial cells. Moreover, LpMACs directly reach into the intestinal lumen with their protrusions (171). The majority of LpMACs are constantly replenished by circulating inflammatory monocytes (Ly6C^hi^) after week 3 of life, i.e. starting with weaning (98). Accordingly, CCR2-deficient mice, which are impaired in monocyte egress from the bone-marrow, are deficient in LpMACs (98, 108). This process is dependent on the microbiota (98).

Once monocytes enter the lamina propria they differentiate and mature into LpMACs, which are characterized by a site specific response program to TLR-stimuli (172). Moreover, maturation of intestinal tolerogenic LpMACs and subsequent tolerance of the gut, similar to the lung, depends on IL-10 and TGFβ signaling (104, 108, 150, 158, 173). Inflammation interferes with this maturation process and leads to the formation of inflammatory effector cells (174), which control neutrophil activation and limit commensal-mediated tissue damage (175). In neonatal mice, an enteral challenge with MCMV-containing milk leads to viral dissemination (176). Yet, adult mice seem largely resistant to this infection mode (11). In HCMV associated intestinal inflammation, CD14^+^ monocytes, the putative human analog of mouse Ly6C^hi^ monocytes (177), upregulate the TGFβ antagonist Smad7, which leads to the acquisition of inflammatory properties of intestinal MACs (178). It is tempting to speculate that early postnatal infections with CMV promote the monocyte influx into the intestine and thus protect against the invasion of commensals and opportunistic pathogens (179).

CMV-infections in fetuses cause severe symptoms and can lead to permanent damage of the CNS with immediate consequences and late sequelae, such as hearing loss. Microglia, the resident MACs of the brain parenchyma, are yolk sac-derived and maintain their population size exclusively via self-renewal (180) (Table 2). Monocyte-derived MACs only populate the brain after blood-brain-barrier disruption (181). In contrast to microglia, CNS-associated MACs include resident tissue MACs of barriers and interfaces of the CNS parenchyma and the periphery, such as perivascular space, meninges and the choroid plexus (154) (Table 2). They are also predominantly embryonically seeded and mainly self-renew (155, 182). Congenital transmission of CMV is best modeled by early postnatal intraperitoneal infection of mice (PND0-2), because a cell-free or cell-associated viremia is preceding focal encephalitis (183, 184) similar to the situation in humans (185). Thus, regions around blood vessels are infected first, including the choroid plexus of the periventricular region (186) and the meninges (187). In these regions MACs identified via F4/80^+^ (186) and Iba1^+^ (ionized calcium-binding adaptor molecule 1) (187) are infected or activated, respectively. In human brain aggregate culture systems, microglia or monocyte-derived MACs also appear to be initially infected (188). From the periventricular region, the meningoencephalitis caused by MCMV spreads to the hippocampus and cortex (186). In mice, MCMV-encephalitis leads to monocyte recruitment, diapedesis and subsequent differentiation into monocyte-derived MACs (189), which were also found to be infected. Thereby, monocyte-derived MACs might represent a potential way of viral dissemination from CNS interfaces into the brain parenchyma (186, 190). Accordingly, infection of MACs in the CNS may lead to heavy reorganization of otherwise tightly regulated immune cell populations, which may contribute to different pathologies (191–193).

Since resident MACs ensure the structural and functional integrity of their respective tissue (194), an exchange with monocyte-derived MACs after postnatal CMV infection bears opportunities, but also risks. On the one hand, tissue resident MACs are terminally differentiated and less plastic compared to monocytes (106). Postnatal infection could induce an early turnover of MACs in tissues like the intestine (98) and more distal, the skin (97). Thereby monocyte-derived MAC may foster maturation of the immune system and change the Th2-biased immune state (138) to a more inflammatory state, which may promote resistance to future infections. On the other hand, in organs, where MACs are largely maintained through self-renewal, invasion of monocytes could lead to exaggerated inflammation and subsequently tissue damage.

## Do CMV infections protect against consecutive challenges or allergy?—Immunomodulation of the innate immune system

Individual immune memory is conventionally attributed to the adaptive immune system. However, it has been known for decades that innate immune cells can be primed by infection for long-lasting alterations in the response to subsequent challenges. These changes in activation programs were variably coined immune priming, immune tolerance, and most recently trained immunity (195–197). As an example for the latter, Rag1^−/−^ mice without T- and B-cells, but not CCR2^−/−^ mice, which are deficient in circulating monocytes, survived a lethal dose of *Candida albicans*, when they had been subjected to a low dose fungal infection one week before (198). Human monocyte and MAC training have been found to involve altered cytokine formation and epigenetic changes (198, 199).

In case of herpesvirus infection models, latent murid herpesvirus 4 (MuHV4) infection leads to the replacement of aMACs with regulatory monocyte-derived MACs, which generate tolerance to house dust mite extracts (200). Furthermore, peritoneal MACs of latently (>28 days) MCMV or mouse gamma-herpesvirus 68 infected mice showed an activated phenotype with increased MHC II expression and a higher killing capacity when re-infected with *Listeria monocytogenes ex vivo*. Latently infected mice were protected against infections with a lethal dose of *L. monocytogenes*. This mode of host resistance was dependent on IFN-γ, but differed from classical IFN-γ-induced protection with respect to both, duration and quality (201).

The fetal and neonatal immune systems have been suggested to be polarized toward protection against extracellular pathogens, which may render them especially vulnerable to viral infections, e.g. by HCMV (138). In contrast, postnatal HCMV infections often pass without overt symptoms and lead to a latent infection with sporadic viral reactivation. It constitutes an attractive model that reactivation occurs, when cues (e.g. interferons) from initial postnatal CMV infection wear off allowing for viral replication (202, 203). In other words, immune priming and recruitment of regulatory myeloid cells may quite rapidly fade. Subsequently, CMV reactivation and containment may induce a further wave of protection/tolerance by innate immune cells without provoking overt disease in immunocompetent individuals. Therefore, infection with CMV may keep the immune system in an alert state, which allows for a rapid response against potentially harmful agents. At the same time, recruitment of regulatory monocytes may maintain tissue integrity and tolerance at mucocutaneous surfaces.

## Myelopoiesis, latency, and reactivation in CMV infections

**Figure 2:**
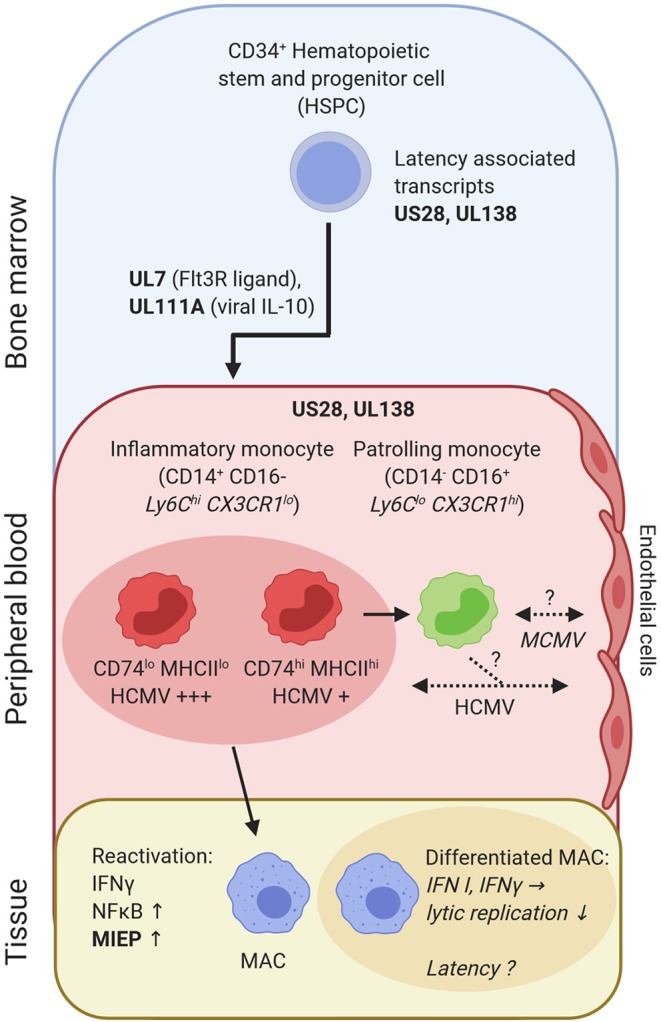
HCMV-derived transcripts (*US28* and *UL138*) are needed to establish latency in CD34^+^ HSPC and CD14^+^ monocytes. HCMV gene products (pUL7, pUL111A) promote preferential differentiation of HSPC into monocytes. CD14^+^ inflammatory monocytes can be subdivided into CD74^hi^ and CD74^lo^ cells, which differ in latent HCMV content and reactivation. Inflammatory monocytes can differentiate into patrolling monocytes. Patrolling monocytes may transfer CMV to uninfected endothelial cells or acquire infection from previously infected endothelial cells [as shown for human CD14^+^ monocytes *in vitro* (204)]. Upon tissue entry and differentiation into monocyte-derived MACs reactivation and lytic replication occurs, once MIEP is activated. IFN-γ and NFκB are central in this process. In contrast, IFN I and IFN-γ protect against active/lytic replication of MCMV in differentiated MACs, which may explain viral latent states observed in MACs. Bold letters, CMV transcripts/genes; italic, mouse data; non-italic, human data; consistent arrow, differentiation; dashed arrow, CMV transmission.

The development along the monocyte-macrophage-axis may be involved in lifelong persistence of CMV (Figure 2). Human CD34^+^ hematopoietic stem and progenitor cells (HSPC) and CD14^+^ monocytes can be latently infected without ongoing replication and virus release (166, 205–209). The proportion of mononuclear cells carrying HCMV genome in latently infected individuals is rather low (1:10^4^-10^5^) (210). Yet, CD14^+^ CD74^lo^ MHCII^lo^ monocytes contain more virus genomes as compared to CD14^+^ CD74^+^ and MHCII^+^ cells (211) (Figure 2). Furthermore, new techniques have allowed for the enrichment and characterization of latency-associated transcripts (212). Expression of US28 and UL138 in HCMV is important to establish latency in HSPC (213–215). Notably, *in vitro* infected HSPC and peripheral blood mononuclear cells (PBMC) from clinical samples showed similar HCMV transcriptome profiles (212), pointing to a potent antiviral program already in immature cells.

The HCMV protein pUL7 binds to the Fms-like tyrosine kinase 3 receptor (Flt3R) and further steers HSPC toward myeloid monocyte commitment (216). Moreover, pUL111A, a viral IL-10 analog, impairs HSPC differentiation into dendritic cells (217) (Figure 2). This is in line with recent single cell sequencing data, where HCMV-infected HSPC predominantly differentiated into monocytes (211). Thus, during the course of infection, viral IL-10 ensures a supply of monocytes, which may aid in HCMV dissemination.

In the absence of acute inflammation, inflammatory monocytes recirculate to the bone marrow (101), where they differentiate into patrolling monocytes (218, 219). Interestingly, inflammatory monocytes are short lived (half-life: 20h in mice; ~1d in humans), yet they control the lifespan of patrolling monocytes (half-life: ~2.2d in mice; ~7.5d in humans) via M-CSF consumption in mice (219, 220). Thus, it seems conceivable that inflammatory monocytes can be infected, harbor CMV and continue to differentiate into patrolling monocytes. This may lead to complex changes in the composition of circulating monocytes. Moreover, infected monocytes could pass CMV on to endothelial cells (204), another cell type discussed for life-long latency in mice (221) and persistent infection in humans (222) (Figure 2). In a mouse model of latency, IFN-β prevents immediate early (IE) gene expression, which confers protection of lytic MCMV replication in endothelial cells. Reactivation of lytic infection occurred, once the effect of IFN-β wore off (203).

Upon activation and differentiation of monocytes into MACs, viral replication can restart (223, 224). *Ex vivo* infection and culturing of primary human CD34^+^ cells until differentiation into MACs, resembling the sequence of myelopoiesis and MAC determination, was associated with HCMV reactivation (225). Transcriptional activation of the major immediate early promoter (MIEP) and subsequent expression of IE1, IE2 (HCMV) or ie1, ie3 (MCMV) genes is a key switch to lytic infection. The enhancer of the MIE locus contains binding sites for NFκB (226). Hence, inflammation and cytokine production, e.g. TNF, may lead to reactivation, which is controlled in immunocompetent individuals. However, in case of an impaired inflammation control, reactivation causes complications as seen in the gut, lung or skin (227–230). Interestingly, in HCMV seropositive individuals IFN-γ producing T-cells appear to be more frequent as in seronegative individuals (231). IFN-γ represents a crucial factor for viral reactivation during the differentiation of human monocyte-derived MACs (224) (Figure 2). However, the presence of IFN-γ also leads to MAC activation and confers protection against lytic MCMV infection in already differentiated MACs (202) (Figure 2).

Early studies suggested that MACs are also a cellular reservoir for viral latency. After administering MCMV into the abdominal cavity, peritoneal MACs were found to bear MCMV DNA 3–9 months after infection. Furthermore, co-culturing with mouse embryonic fibroblasts resulted in reactivation of lytic viral replication, arguing for latently infected MACs (232). Another study used PCR *in situ* hybridization (PISH) to label viral DNA in tissue sections 6 months after peritoneal infection with MCMV. LAMP-2^+^ (CD107b^+^/Mac-3^+^) bona fide lung MACs were found to carry MCMV genome. However, the association of MCMV PISH- and LAMP-2-positivity were based on colocalisation in interalveolar tissue and not determined on the single cell level, which hampers the interpretation of these data (233).

In summary, CMV latency in myeloid cells may provide solutions for several puzzles in CMV disease progression and control, yet further data are required to robustly establish this scenario. In particular the discrimination between human inflammatory/classical and patrolling/non-classical monocytes could serve well to translate murine *in vivo* models into the human system.

## Conclusions

In CMV infection, barrier tissue MACs are both targets and effector cells. The early formation of antiviral IFNs, which control several thousand of genes (234), is essential for regulating the immune response. The expression of numerous IFN inhibitory proteins by both HCMV and MCMV (235) is in full support of a model, where the armament of host and virus ultimately serves both sides. Subsequent signaling events, including the formation of IL-10, impact on restricting CMV-induced immunopathology and antiviral immunity, thus allowing for reestablishment of tissue immune homeostasis, as well as viral latency for years. When CMV infection occurs very early in life, as it has in most of human history, antiviral immunity and individual development of myeloid cells are intertwined. This is particular true for organs with high turnover of MACs, since monocytes as MAC progenitors integrate cues from CMV into the site specific differentiation program. Accordingly, in the case of an immunocompetent host, CMV and tissue MACs are primarily not foes. On the contrary, given the ancient CMV adaptation to mammalian hosts, it is a relationship with reciprocal benefits, e.g. the tuning of basal activation for a better response against more harmful microbial invaders, the renewal of tissue resident cells and modulation of autoimmunity (as it has been shown for gamma herpesviruses). At the same time, CMV has developed strategies to manipulate host immunity for lifelong persistence and inter-individual spread. Therefore, adverse consequences of CMV in the elderly, e.g. T-cell inflation (236) may be due to a CMV-human co-evolution tailored for a shorter host lifespan. Currently, direct evidence for such “mutual friendship” is just emerging. Yet, a scenario, where the benefits and harms of CMV infection are tissue and context specific, is highly attractive.

## Author Contributions

SB, ZR, and PH wrote and edited the manuscript.

## Conflict of Interest

The authors declare that the research was conducted in the absence of any commercial or financial relationships that could be construed as a potential conflict of interest.
